# A Novel Antiplatelet Aggregation Target of Justicidin B Obtained From *Rostellularia Procumbens* (L.) Nees

**DOI:** 10.3389/fphar.2019.00688

**Published:** 2019-06-14

**Authors:** Yan-Fang Yang, Song-Tao Wu, Bo Liu, Zhou-Tao Xie, Wei-Chen Xiong, Peng-Fei Hao, Wen-Ping Xiao, Yuan Sun, Zhong-Zhu Ai, Peng-Tao You, He-Zhen Wu

**Affiliations:** ^1^Faculty of Pharmacy, Hubei University of Chinese Medicine, Wuhan, China; ^2^Key Laboratory of Traditional Chinese Medicine Resources and Chemistry of Hubei Province, Wuhan, China; ^3^Collaborative Innovation Center of Traditional Chinese Medicine of New Products for Geriatrics Hubei Province, Wuhan, China

**Keywords:** justicidin B, integrin α_IIb_β_3_, platelet aggregation, gene chip, LC-MS, network pharmacology, Prometheus NT.48, microscale thermophoresis

## Abstract

The present study explored the possible bioactive ingredients and target protein of *Rostellularia procumbens* (L.) Nees. Firstly, we found that the ethyl acetate extraction obtained from *R. procumbens* could inhibit platelet aggregation. Then, gene chip was used to investigate differentially expressed genes and blood absorption compounds were investigated using high performance liquid chromatography-mass spectrometry characterization (LC-MS). Depending on the results of gene chip and LC-MS, the targets of blood absorption compounds were predicted according to the reverse pharmacophore matching model. The platelet aggregation-related genes were discovered in databases, and antiplatelet aggregation-related gene targets were selected through comparison. The functions of target genes and related pathways were analyzed and screened using the DAVID database, and the network was constructed using Cytoscape software. We found that integrin α_IIb_β_3_ had a highest degree, and it was almost the intersection of all pathways. Then, blood absorption compounds were screened by optical turbidimetry. Western blot (WB) revealed that justicidin B separated from the ethyl acetate fraction may inhibit the expression of integrin α_IIb_β_3_ protein. For the first time, we used Prometheus NT.48 and MST to detect the stability of this membrane protein to optimize the buffer and studied the interaction of justicidin B with its target protein. To our best knowledge, this is the first report to state that justicidin B targets the integrin α_IIb_β_3_ protein. We believe that our findings can provide a novel target protein for the further understanding of the mechanism of *R. procumbens* on platelet aggregation.

## Introduction

Integrin α_IIb_β_3_ is a major platelet-surface receptor for the regulation of platelet aggregation and thrombosis. Fibrinogen is attached to the integrin α_IIb_β_3_ protein of one platelet and is linked to the integrin α_IIb_β_3_ protein of another platelet. At the same time, the platelets are grouped together to cause a platelet aggregation cascade. Therefore, integrin α_IIb_β_3_ is a key protein for platelet aggregation (Coller and Shattil, 2008; Yun et al., 2016; Andrews and Gardiner, 2017; Bender et al., 2017; Chatterjee and Gawaz, 2017).

*Rostellularia procumbens* (L.) Nees (Acanthaceae) is widely distributed in the Taiwan Province and the southwest provinces and has been proven to have a huge potential for the development of Chinese medicine owing to its plant resources, chemical constituents, pharmacological action, and clinical application. It has complex chemical composition (Savithramma et al., 2007; Joshi and Joshi, 2000; Committee, 2011).

In addition, reports have shown that *R. procumbens* has significant pharmacological properties such as anti-viral and anti-tumoral (Chen et al., 1995; Corrêa and Alcântara, 2012). It has also been noted that aqueous extracts of *R. procumbens* decrease platelet aggregation (Chen et al., 1996). According to preliminary experiments, ethyl acetate extract is the active fraction. Animal experimentation conducted analyze this fraction. Then, gene chip was used to investigate differentially expressed genes. Blood absorption compounds were investigated using LC-MS. Targets of blood absorption compounds were predicted according to the reverse pharmacophore matching model. The platelet aggregation-related genes were found in databases, and antiplatelet aggregation-related gene targets were selected through comparison. The functions of target genes and related pathways were analyzed and screened using the DAVID database, and the network of antiplatelet aggregation effect of blood absorption compounds was constructed using Cytoscape software. However, the detailed molecular interaction between justicidin B and its target is still unknown. Firstly, blood absorption compounds were screened by optical turbidimetry. Prometheus NT.48 is used to detect protein stability and screen buffer (Maschberger et al.). Then, we compared the two models of microscale thermophoresis (MST) and used NT.115 to verify the interaction between the compound and the integrin α_IIb_β_3_ protein (van den Bogaart et al., 2012; Batoulis et al., 2016; Sparks and Fratti, 2019; Vinothkannan Ravichandran et al., 2018).

In this study, according to preliminary experiments, blood absorption compounds of *R. procumbens* were screened by serum pharmacological. Gene chip and network pharmacology were used to find the target. Then, the interaction between justicidin B and the membrane protein integrin α_IIb_β_3_ was verified by Western blot (WB) and MST. It would lay the groundwork for understanding the molecular mechanism involved in the inhibition of platelet aggregation by *R. procumbens*.

## Materials and Methods

### Chemical and Materials

The ethyl acetate extract was obtained from the Key Laboratory for Traditional Chinese Medicine Resources and Chemistry of Hubei Province (Chen, 2012; Su, 2014; Wu et al., 2013) [ethyl acetate (EtOAc), Lichrosolv, purity ≥ 99.9%]. Acetonitrile (ACN) was of LC-MS grade and was purchased from Thermo-Fisher (Pittsburgh, PA, USA). Deionized water was made available in-lab using a Milli-Q purification instrument (Millipore, Bedford, MA, USA). 5-Hydroxytryptophan was obtained from National Institutes for Food and Drug Control (China). Thrombin was purchased from BioMed Lublin, Poland. Arachidonic acid (AA), bovine serum albumin (BSA), adenosine diphosphate (ADP), β-acetyl-γ-O-hexadecyl-L-α-phospharidylcholine hydrate (PAF), 12-O-tetradecanoylphorbol-13-acetate (PMA), and dimethyl sulfoxide (DMSO) were purchased from Sigma (St. Louis, MO, USA). RNeasy Mini Kit and RNase-free DNase I were purchased from QIAGEN. Pico Reagent Kit, GeneChip90 Hybridization, Wash, and Stain Kit were purchased from Affymetrix. Integrin α_IIb_β_3_ monoclonal antibody was purchased from SAB (Maryland, USA), and β-actin and anti-mouse IgG were purchased from Cell Signaling (Boston, USA).

### Experimental Animals

SD male rats (190–230 g) were randomly divided into two groups of 16 animals per group, treated orally as follows: control group received 0.5% CMC-Na, groups of ethyl acetate extract (97.20 g/ml) (fasting for 12 h before intragastric administration, administered by intragastric administration at 1 ml/200 mg twice a day for 3 days). After the last administration for 1.5 h, we took 5–8 ml of blood from the femoral artery. All experimental procedures were approved by Animal Care and Use Committee of Institute of Materia Medica, People’s Republic of China.

### Extraction and Detection of Total RNA in Two Groups of Platelets

The platelet of the control group and ethyl acetate group at 4°C and 12,000 rpm was centrifuged for 10 min using RNeasy Mini Kit separation of total RNA. The RNA concentration and A260/A280 ratio were determined using an SMA 3000 microspectrophotometer (Meriton, China). The results showed that the ratio was 2.00:2.05, indicating a high purity of the extracted RNA, which was deemed suitable for subsequent analysis. After this, a total of 1 µg RNA was used for 1% agarose gel electrophoresis. The ratio of 28S/18S was then determined using a JS-380A Bioanalyzer (Shanghai, China) to determine the quality of RNA. If the RNA integrity number (RIN) > 7.0 and 28S/18S > 0.7, samples were transcribed by a Pico Reagent Kit. Following this, samples were prepared using a GeneChip90 Hybridization, Wash, and Stain Kit (Affymetrix, Thermo Fisher Scientific, Inc.). Then, chips were scanned using a GeneChip Scanner 3000 (Affymetrix, Thermo Fisher Scientific, Inc.).

### Detection of Blood Absorption Compounds

Justicidin B, chinensinaphthol methyl ether, and 6’-hydroxy justicidin B were weighed accurately, and 1 mg/ml control solution with methanol was prepared. The control solution was stored at 4°C for use. The supernatant that was treated by solid phase microextraction cartridge was collected and dried by nitrogen after centrifugation (12,000 r/min, 10 min). Before the liquid injection, 200 μl acetonitrile was used for redissolution and the supernatant was determined by HPLC-DAD-ESI-MS after filtration.

The chromatography analysis was performed on an Agilent XDB-C18 column (150 mm × 4.6 mm, 4 µm) at 25°C; the mobile phase was a mixture of aqueous solutions containing water (A) and acetonitrile (B). The gradient elution procedure was made as follows: 0–14 min, 26% B; 14–51 min, 26–38.5% B; 51–80 min, 38.5% B. The injection volume was 100 μl, and the flow rate was 1 ml/min. The wavelength of 190–690 nm was used for the detection. Mass spectrometry detection was set as follows: capillary temperature, 200°C; source voltage, 4.5 kV for the positive ion mode. The mass range was from 50 to 1,600. Blood absorption compounds were identified by accurate mass, MS/MS ion fragment pattern, and retention time of LC and then were validated by available standard.

### Prediction and Analysis of Differentially Expressed Target Genes

Based on the results of the gene chip and LC-MS, we conducted a network pharmacology study. The PharmMapper Server (http://lilab.ecust.edu.cn/pharmmapper/index.php) is a freely accessed web server used to identify potential target candidates for given probe blood absorption compounds using a pharmacophore mapping approach. GeneCards (https://www.genecards.org/) and MalaCards (http://www.malacards.org/) were used for potential target screening from the results of gene chip. All potential target genes were collected and uploaded to the DAVID (https://david.ncifcrf.gov/summary.jsp) database. Following this, the functions and signaling pathways of target genes as well as pathway enrichment were investigated *via* Gene Ontology (GO; http://geneontology.org/) and Kyoto Encyclopedia of Genes and Genomes (KEGG) pathway analyses (http://www.genome.jp/kegg/ko.html).

### Construction of the Network

The blood absorption compounds and target genes were imported into Cytoscape 3.6.1 software to build an active compound/target gene/pathway network and an active compound/platelet aggregation-related target gene network. The blood absorption compounds and target genes were input as the node. If there was a connection between two nodes, edge was used to show the connection.

The network was then analyzed with the network analyze function. High degree gene targets in the protein interaction network were analyzed. According to the results of KEGG enrichment, the pathways with higher counts were selected to analyze their key targets. Meanwhile, the genes suitable for the analysis of the targets were obtained through comparative analysis from the literature and database.

### Screening of Active Compounds Inhibiting Platelet Aggregation

Platelet-rich plasma (PRP) was prepared by centrifugation of fresh blood at 200×*g* for 10 min at room temperature and aspirating the supernatant. Platelet-poor plasma was then sedimented by centrifugation of residual blood at 800×*g* for 10 min at room temperature. Blood platelet aggregation was monitored by platelet turbidity, with 0% aggregation calibrated as the absorbance of platelet-poor plasma and 100% aggregation as the absorbance of PRP. PRP was incubated with the drug at 37°C for 20 min and then stimulated with AA, ADP, thrombin, PAF, and PMA. The aggregation of PRP (preincubated with the tested plant fraction) in response to 10 µM inducer was recorded using an aggregometer (LBY-NJ4).

### Western Blotting to Detect Integrin α_IIb_β_3_

Just like 2.7, we got PRP what were split into four groups: blank control group, inducer group, justicidin B induction group, and the last group received aspirin. Different groups were cultured 1.5 h, and the incubator was set to 5% CO_2_ at 37°C. Then, platelets were washed with PBS twice, harvested, and lysed in RIPA buffer containing protease inhibitors on ice for 60 min. The protein concentration was determined by the BCA method. Next, protein samples (18 μg) were equally loaded onto SDS-PAGE and electrotransferred to PVDF membranes. Subsequently, the membranes were blocked with 5% non-fat milk for 1 h and incubated overnight with primary antibodies. The membranes were washed with TBST buffer three times and then incubated with secondary antibodies for 1 h at 25°C. The membranes were then rinsed three times with blocking solution and visualized by the ECL detection system.

### NT. LabelFree Analysis

Since integrin α_IIb_β_3_ is a membrane protein, we used NT. LabelFree to prevent the label from affecting the results. Titration series with ligand concentrations varying between 0 and 1,000 mM were prepared in the Prometheus NT.48 optimized buffer (**Figure S1**). SD-test (the automatic pre-detection of MST to detect the stability of protein signal) was required before measurement.

Approximately, 3 µl was loaded into NT.LabelFree standard-treated capillaries (Nanotemper). MST experiments were performed at 40% MST (infra-red laser) power and 60% LED power at 25°C using the Monolith NT.LabelFree Instrument (Nanotemper). Ratios between normalized initial fluorescence and after temperature-jump and thermophoresis were calculated and averaged from five to nine independent runs. Means of fluorescence intensity obtained by the MST measurements were fitted, and the resultant Kd values were given together with an error estimation from the fit by the built-in formula of the analysis software.

### NT.115 Analysis

The fluorescence of the ligand interfered with the result. This was further exacerbated when using label-free thermophoresis owing to the additional noise present in measuring fluorescence in ranges where inherent fluorescence of the protein itself is measured. After the SD-test experiment verified that the label had little effect on the protein, we used NT.115 for measurement.

All the compounds were analyzed with the concentration gradient of 50 µM with 20 µM of CviR that was labeled Monolith NTTM Protein Labeling Kit RED–NHS (Cat Nr: L001) before instrumental analysis. LED power was 20% and Prometheus NT.48 optimized buffer was used only for the analysis.

Analysis was performed on Monolith Nano Temper (NT)115 and its accessory, i.e., standard-treated 4-µl volume glass capillaries were employed to measure the molecular interaction (Nano Temper Technologies GmbH, Munich, Germany). Means of fluorescence intensity obtained by the MST measurements were fitted, and the resultant Kd values were given together with an error estimation from the fit by the built-in formula of NT1.5.41 analysis software.

## Results

### Microarray Results

Compared with the control group, hundreds of genes were revealed to be differentially expressed in platelet in EtOAc extract group (**Table 1**). In EtOAc extract group, PLCB2, PRKCA, GNAQ, MAPK10, MAPK8, MAPK11, MAPK14, GNAI2, PIK3CG, and PIK3R1 were markedly down-regulated in the model group compared with the control group.

**Table 1:** The top different 50 genes of platelet expression between the ethyl acetate extract group and blank group.

Rank	Gene symbol	Gene annotation
1	PRKACG	Protein kinase, cAMP-dependent, catalytic, gamma
2	PRKCA	Protein kinase C, alpha
3	ADCY5	Adenylate cyclase 5
4	PLCB2	Phospholipase C, beta 2
5	PLCB4	Phospholipase C, beta 4
6	NFKB1	Nuclear factor of kappa light polypeptide gene enhancer in B-cells 1
7	TJP1	Tight junction protein 1
8	CTNNB1	Catenin (cadherin-associated protein), beta 1
9	ENPP3	Ectonucleotide pyrophosphatase/phosphodiesterase 3
10	GLB1	Galactosidase, beta 1
11	GJA1	Gap junction protein, alpha 1
12	ADCY4	Adenylate cyclase 4
13	CYP2C9	Cytochrome P450, family 2, subfamily C, polypeptide 9
14	CDC42	Cell division cycle 42
15	MAPK10	Mitogen-activated protein kinase 10
16	MAPK8	Mitogen-activated protein kinase 8
17	CYP4A11	Cytochrome P450, family 4, subfamily A, polypeptide 11
18	PIK3CG	Phosphoinositide-3-kinase, catalytic, gamma polypeptide
19	NT5E	5’-Nucleotidase, ecto
20	PTGS1	Prostaglandin-endoperoxide synthase 1
21	STAT1	Signal transducer and activator of transcription 1
22	PIK3R1	Phosphoinositide-3-kinase, regulatory subunit 1
23	PLD2	Phospholipase D2
24	PIK3CD	Phosphoinositide-3-kinase, catalytic, delta polypeptide
25	MAOB	Monoamine oxidase B
26	GSK3B	Glycogen synthase kinase 3 beta
27	MAPK11	Mitogen-activated protein kinase 11
28	MAPK14	Mitogen-activated protein kinase 14
29	EGFR	Epidermal growth factor receptor
30	VEGFA	Vascular endothelial growth factor A
31	ATF4	Activating transcription factor 4
32	CRKL	v-crk sarcoma virus CT10 oncogene homolog
33	PPP2CB	Protein phosphatase 2, catalytic subunit, beta isozyme
34	PPP2R1A	Protein phosphatase 2, regulatory subunit A, alpha
35	GBA	Glucosidase, beta, acid
36	IMPAD1	Inositol monophosphatase domain containing 1
37	HK2	Hexokinase 2
38	HK3	Hexokinase 3
39	CYP3A4	Cytochrome P450, family 3, subfamily A, polypeptide 4
40	ITGB2	Integrin, beta 2 (complement component 3 receptor 3 and 4 subunit)
41	UGT2B7	UDP glucuronosyltransferase 2 family, polypeptide B7
42	SMAD3	SMAD family member 3
43	RAF1	cdna:known gene:ENSG00000132155
44	CYP2B6	Cytochrome P450, family 2, subfamily B, polypeptide 6
45	PLB1	Phospholipase B1
46	ACAA2	Acetyl-CoA acyltransferase 2
47	GNAI1	G protein, alpha inhibiting activity polypeptide 1
48	GNAI2	G protein, alpha inhibiting activity polypeptide 2
49	ACAA2	Acetyl-CoA acyltransferase 2
50	GNAQ	G protein, alpha q polypeptide

### The Blood Absorptions Compounds of EtOAc Extract

**Figure 1:**
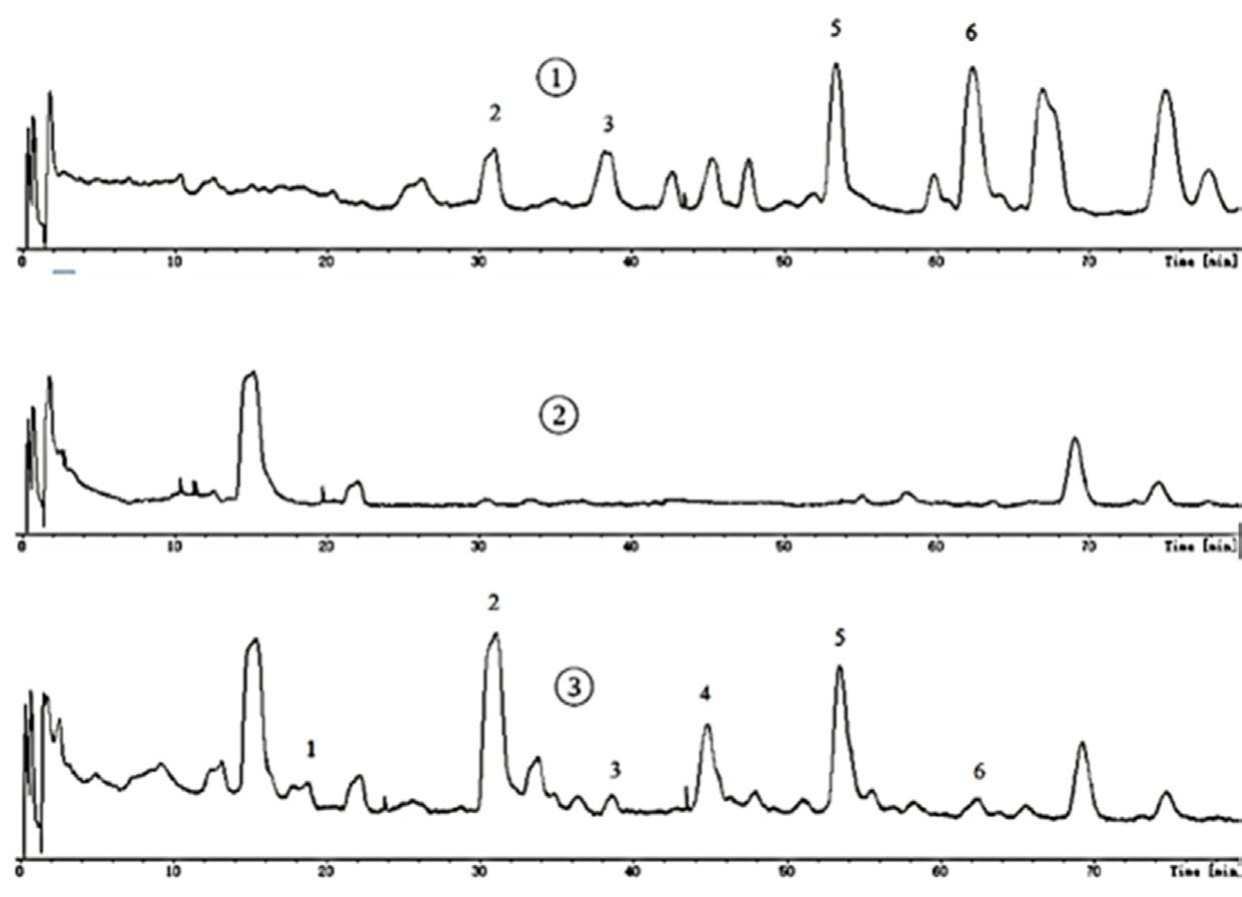
Mass spectrometry.

All samples were analyzed by LC-MS according to chromatographic conditions. By comparing the relative retention time and UV absorption spectra of the chromatographic peaks of the test solution and the mixed control solution, the information of the mass spectrometry fragmentation, combined with the preliminary research results and literature of the laboratory, we can confirm that 6’-hydroxy justicidin B, justicidin B, and chinensinaphthol methyl ether are the prototype compounds of the ethyl acetate extract in the blood (**Figure 1**, **Table 2**).

**Table 2:** HPLC-DAD-ESI-MS data and identification of the compounds *in vitro* and *in vivo*.

Peak	TR/min	MW	Molecular formula	*In vitro* compounds (*m*/*z*)	Blood absorptions compounds (*m*/*z*)	Identification
1	18.8	352	C_20_H_16_O_6_	—	353.1012, 335.0896, 325.0494, 307.0914	ProcumbenosideL conversion compound
2	31.0	380	C_21_H_16_O_7_	381.0975,363.0875, 337.1063,279.0969, 137.0161	381.1050, 363.0880, 337.1122, 279.0973, 137.0189	6’-Hydroxy justicidin B
3	38.6	410	C_22_H_18_O_8_	411.1084,393.0962, 274.0833, 137.0189	411.1064, 393.0958, 274.0819, 137.0177	6’-Hydroxy justicidin A
4	44.8	410	C_22_H_18_O_8_	433.0881,411.1051, 393.0939,381.0966, 244.5356	433.0878, 411.1049, 393.1009, 381.0866, 244.5389	6’-Hydroxy justicidin C
5	53.4	364	C_21_H_16_O_6_	365.1105,335.0881, 321.1084, 291.0983	365.1105, 335.0881, 321.1084, 291.0983	Justicidin B
6	62.4	394	C_22_H_18_O_7_	395.1194,381.0905, 365.0982,351.1203, 244.0503	417.0942, 395.1110, 381.1024, 351.1185, 244.0505	Chinensinaphthol methyl ether

### Prediction and Analysis of the Target Genes

**Figure 2:**
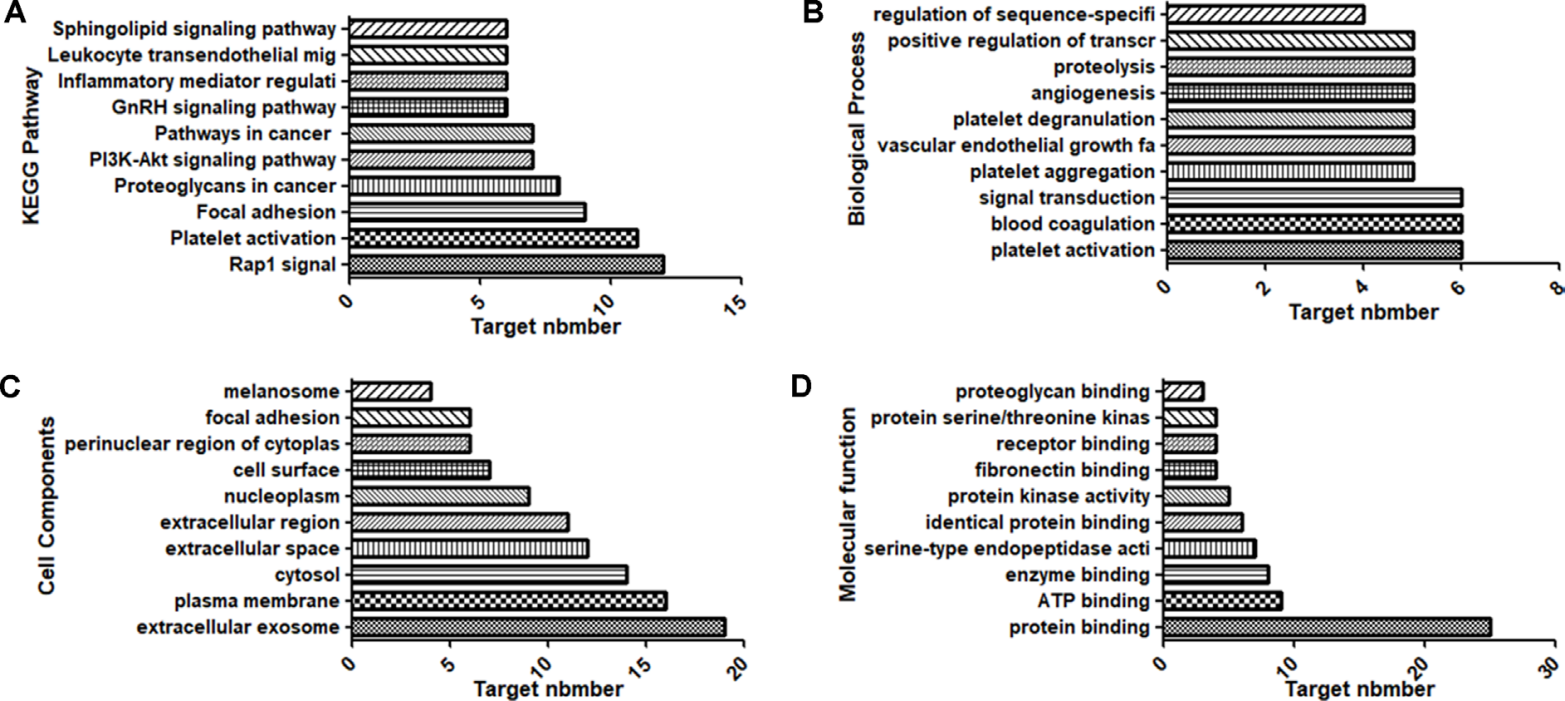
Top 10 components of the KEGG pathway and GO enrichment analyses. **(A)** KEGG pathways. **(B)** Biological process. **(C)** Cell components. **(D)** Molecular function.

All potential target genes were synthesized and uploaded to the DAVID database for KEGG pathway annotation and GO enrichment. The threshold was set as P ≤ 0.05, and the pathways or gene functions with higher count were analyzed. The top 10 pathways were graphed by GraphPad Prism 6 (**Figure 2**).

KEGG pathway annotation showed that 30 of the 31 potential target genes were enriched (96.8%) and involved 62 pathways, and 27 of these pathways were significantly correlated with the target genes (P ≤ 0.05). The following pathways had the largest number of genes involved: Platelet activation (11, 35.5%), Rap1 signaling pathway (12, 38.7%), Focal adhesion (9, 29.0%), Proteoglycans in cancer (8, 25.8%), PI3K-Akt signaling pathway (7, 22.6%), and Pathways in cancer (7, 22.6%). GO enrichment analysis showed that the number of genes involved in the CC (Cell Components), MF (Molecular Function), and BP (Biological Process) targets was 31 (100%). CC enrichment was mainly involved in following target genes: extracellular exosome (19, 61.3%), plasma membrane (16, 51.6%), and cytosol (14, 45.2%). MF enrichment was mainly involved in the following target genes: protein binding (25, 80.6%), ATP binding (9, 29.0%), and enzyme binding (8, 25.8%). BP enrichment was mainly involved in the following target genes: platelet aggregation (5, 16.1%), platelet activation (6, 19.4%), and blood coagulation (6, 19.4%).

### Construction of the Network

**Figure 3:**
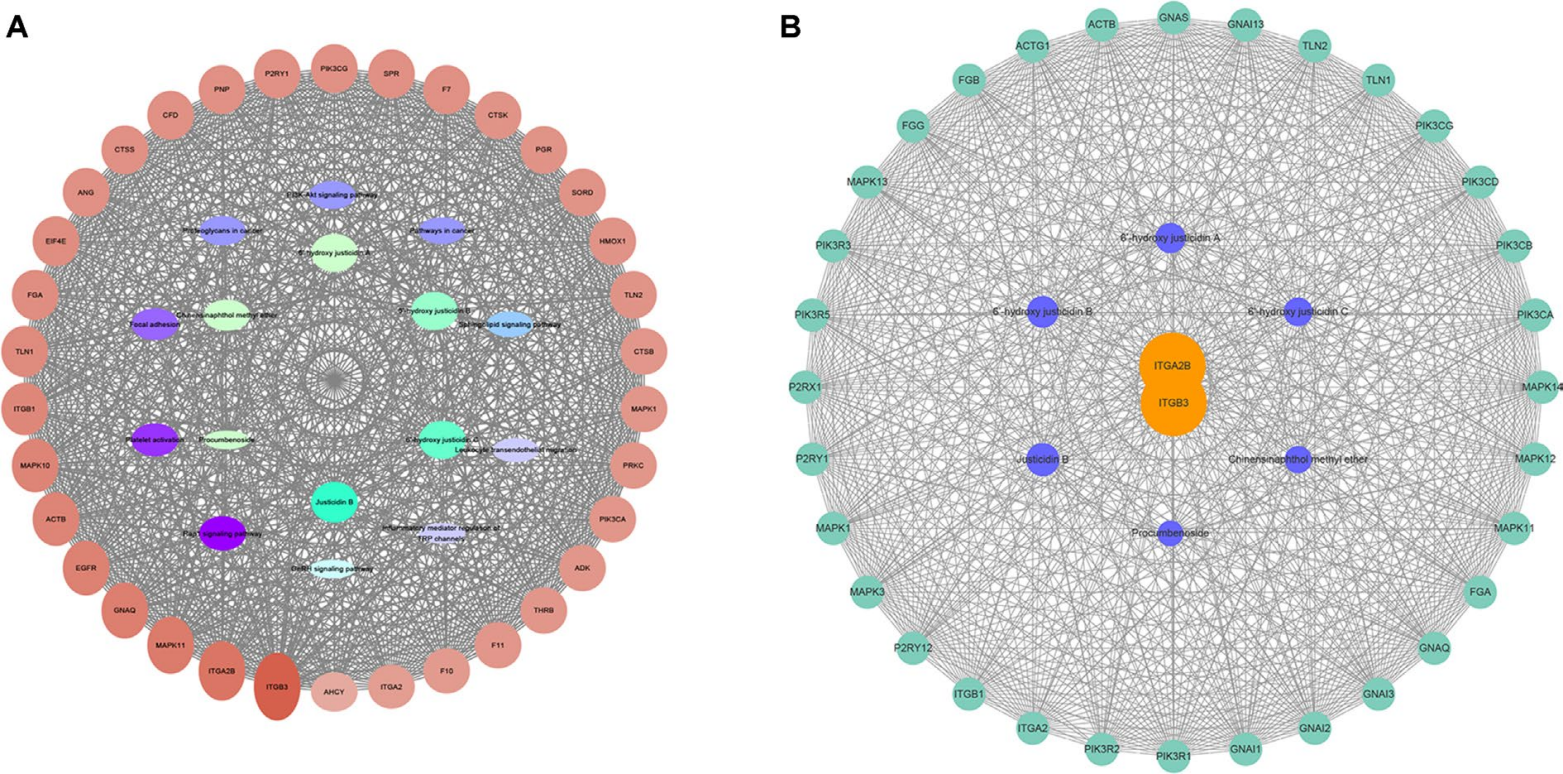
Network diagram constructed by Cytoscape. **(A)** Network diagram of active components/target genes/enrichment pathways. **(B)** Network diagram of active components/platelet aggregation-related target genes.

According to the results of gene chip, target genes in the top 10 pathways and compounds were selected to construct an active compound/target gene/pathway network and an active compound/platelet aggregation-related target gene network (**Figure 3**). The network diagram shows the synergistic effect of various compounds on multiple targets when* R. procumbens* plays a role in antiplatelet aggregation effects.

The network analyze tool was used to analyze the network, and genes with higher degree were associated with more genes. It can be considered that the corresponding protein of the genes plays an important role in central correlation when *R. procumbens* plays an antiplatelet aggregation role. The integrin α_II_bβ3 (integrin α_II_bβ3 is a protein that in humans is encoded by the ITGA2B and ITGB3 gene) had the highest degree; it was the intersection of all platelet-related pathways.

The results were compared with those of the KEGG pathway analysis and combined with the literature study. Integrin α_IIb_β_3_ was selected as the target for the binding validation experiment.

### Screening of Active Compounds

**Figure 4:**
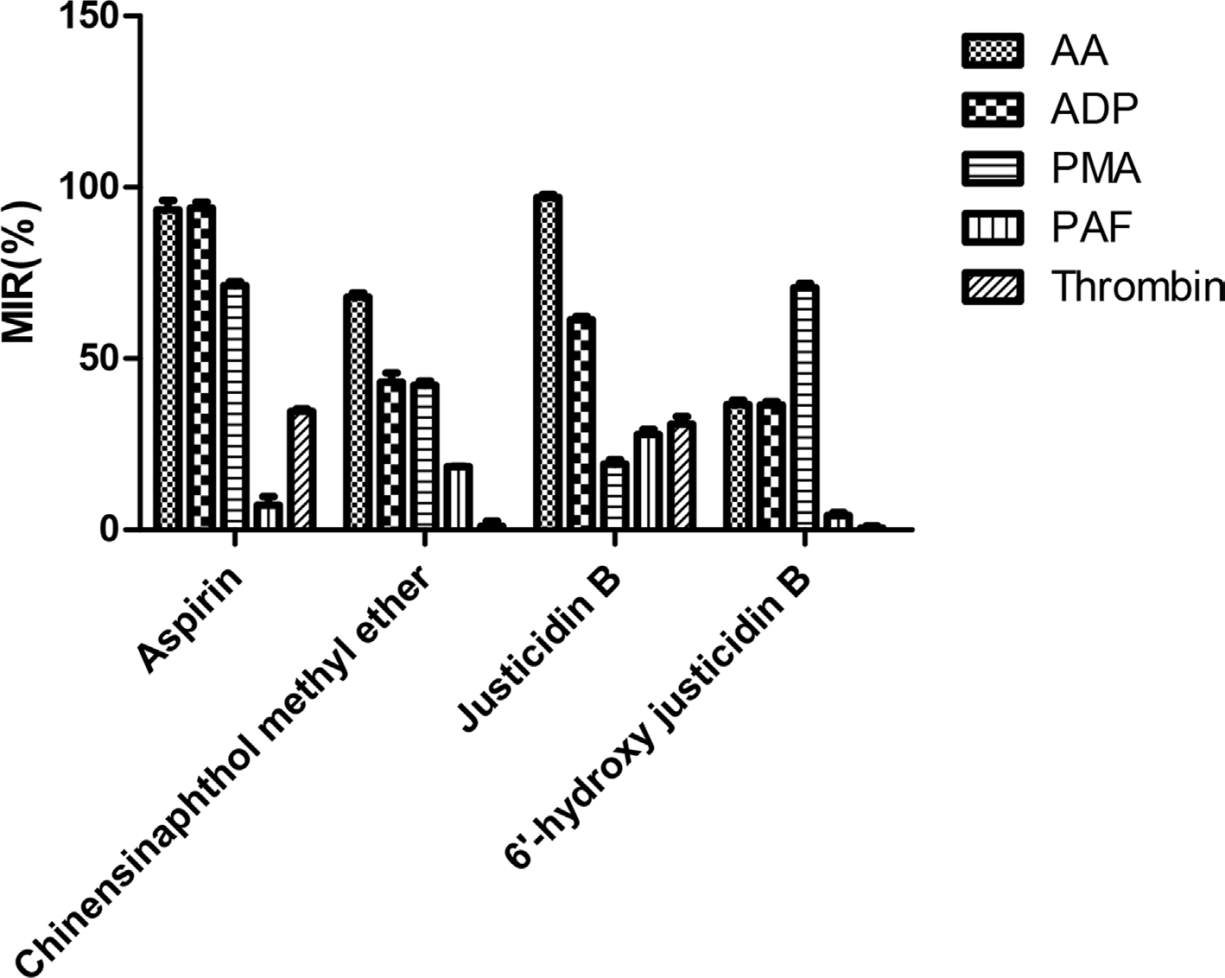
MIR (%) of different compounds.

Platelet with four kinds of inducers was stimulated, and platelet aggregation was detected after being incubated by these isolated blood absorption compounds. The platelet aggregation inhibition rate (MIR) was calculated to indicate the inhibitory activity of the drug. Results showed that those justicidin B groups had higher maximum aggregation compared with others. The remaining compounds showed different degrees of antiplatelet aggregation (**Table 3**, **Figure 4**).

**Table 3:** MIR (%) of different compounds.

Compounds (x ± SD, n = 3)	MIR (%)				
	AA	ADP	PMA	PAF	Thrombin
Aspirin	93.43 ± 2.81	93.88 ± 1.75	71.26 ± 1.21	7.22 ± 2.51	34.59 ± 0.77
Chinensinaphthol methyl ether	67.94 ± 1.27	43.12 ± 2.74	42.22 ± 1.16	18.40 ± 0.12	1.04 ± 1.51
Justicidin B	97.04 ± 0.85	61.26 ± 1.05	19.18 ± 1.28	27.87 ± 1.39	30.76 ± 2.27
6’-Hydroxy justicidin B	36.53 ± 1.29	36.46 ± 1.01	70.69 ± 1.25	4.13 ± 0.95	0.50 ± 0.74

### Integrin α_IIb_β_3_ Expression in PRP

**Figure 5:**
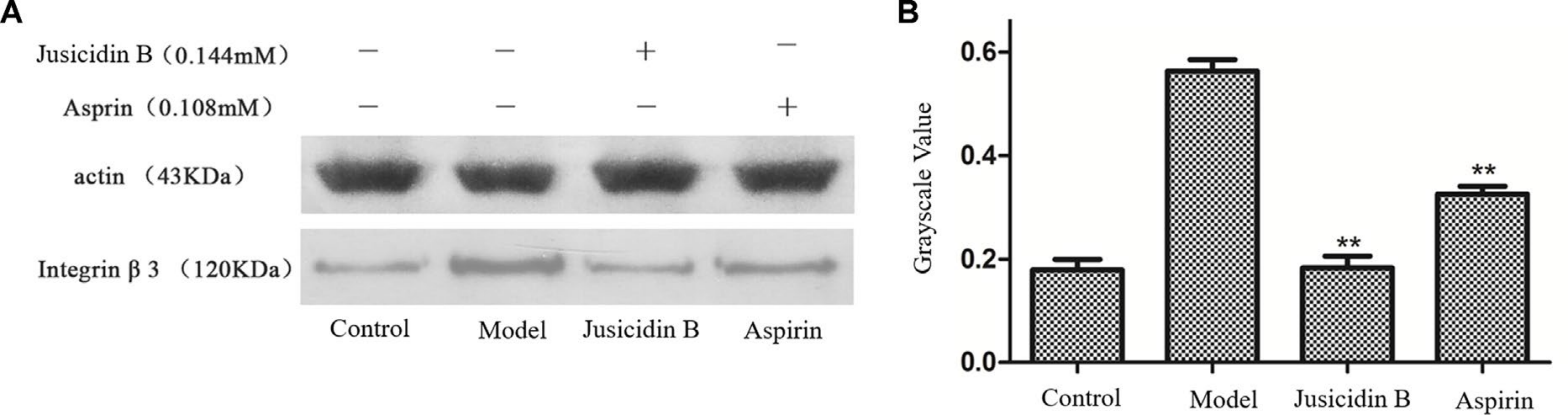
The influence of justicidin B on integrin α_IIb_β_3_ protein. **(A)** Western blot. **(B)** Grayscale value.

We investigated whether justicidin B had a regulatory effect on integrin α_IIb_β_3_ expression. As shown in **Figure 4**, the integrin α_IIb_β_3_ level was significantly increased on the inducer group (P < 0.01, versus control group). More remarkably, justicidin B produced a dramatically inhibited effect on integrin α_IIb_β_3_ level (versus inducer group, P < 0.01, **Figure 5**).

### NT. LabelFree

**Figure 6:**
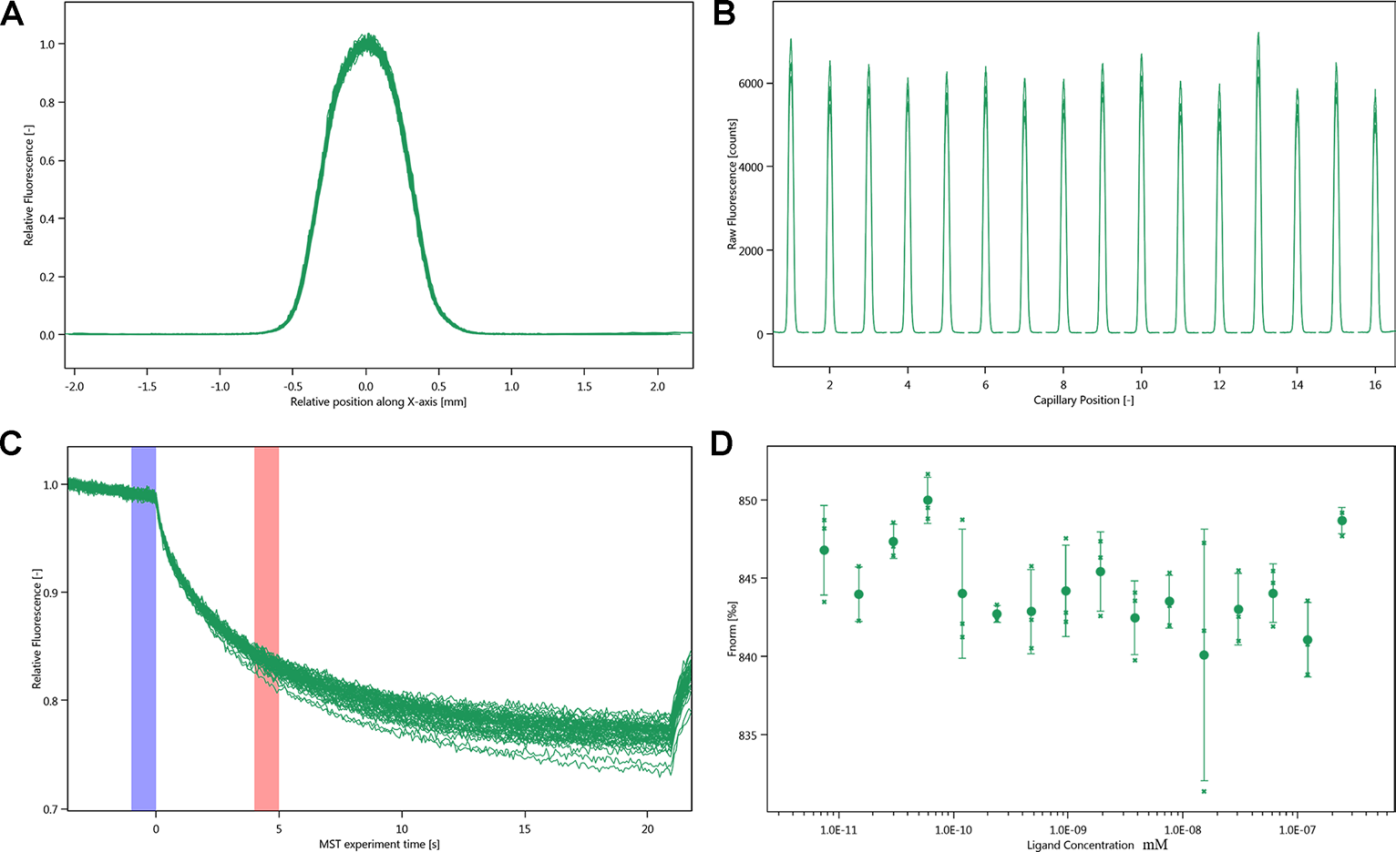
Molecular interaction of integrin α_IIb_β_3_ using NT. LabelFree analysis. **(A)** SD-TEST capillary shap. **(B)** SD-TEST capillary scan. **(C)** MST time traces of 16 different justicin B concentrations (ranging from 0.00748 to 245 µM). **(D)** Dependence of the MST signal on the justicin B concentration (measured 30 s after turning on heating; data from **C**).

MST experiments were performed to detect the molecular interaction between inhibitor and integrin α_IIb_β_3_. Owing to the influence of ligand fluorescence, we obtained unsatisfactory results under the SD-test pass condition, accompanied by large errors (**Figure 6**).

### NT.115

**Figure 7:**
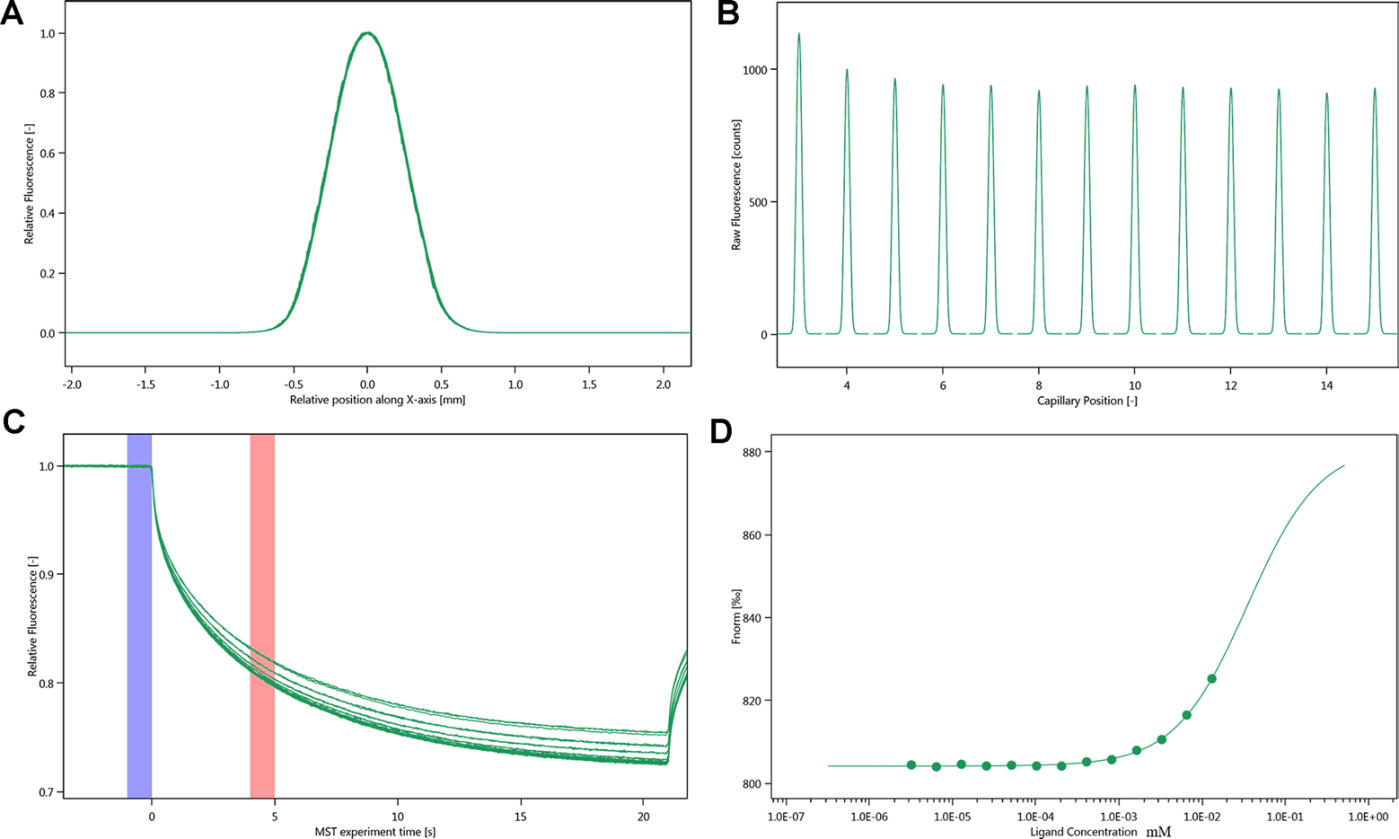
Molecular interaction of integrin α_IIb_β_3_ using NT. 115 analysis. **(A)** SD-TEST capillary shap. **(B)** SD-TEST capillary scan. **(C)** MST time traces of 16 different justicin B concentrations (ranging from 0.0032 to 13.1 mM). **(D)** Dependence of the MST signal on the justicin B concentration (measured 30 s after turning on heating; data from **C**).

The SD-test verified that the label had less effect on the protein. We used NT.115 for the experiment. As differences in normalized fluorescence of the bound and unbound state allow determination of the fraction bound, the dissociation constant was thus calculated. All values were multiplied by a factor of 1,000, which yielded the relative fluorescence change in per thousand. The Kd values of justicin B were 35.017 ± 5.9014 μm (**Figure 7**).

## Discussion

*R. procumbens* has been used in herbal medicines for promoting blood circulation and pain relief. Modern pharmacological studies have shown that *R. procumbens* has a good antiplatelet aggregation effect (Chen et al., 1996). According to preliminary experiments, ethyl acetate extract is the active fraction. However, the chemical composition of this plant material is quite complex. Therefore, it was difficult to determine the active ingredient and target protein by using traditional methods.

The animal experiment was utilized for further verification. Results of gene chip illustrated that ethyl acetate extract could inhibit Gq–PLC–PKC pathway and Gi–PI3K–MAPK pathway. The down-regulation of GNAI1 gene related to the regulation of AC kinase was also observed, indicating that the ethyl acetate site can reduce inhibitory effect of Gi and enhance the activity of AC kinase. This result indicated that the ethyl acetate site could regulate the GI–AC–CAMP signaling pathway. Above all, ethyl acetate might inhibit platelet aggregation by inhibiting Gq–PLC–PKC, Gi–PI3K–MAPK, and other signaling pathways.

In order to further explore the material basis of* R. procumbens*, the blood absorption compounds of *R. procumbens* were screened by serum pharmacological method. Based on the results of the gene chip and LC-MS, GO enrichment analysis found that the targets involved extracellular exosome, plasma membrane, extracellular space, cytosol, and other cell compartments. At the molecular level, the targets were involved in protein binding, enzyme binding, ATP binding, and other molecular activities, and they were related to platelet aggregation, platelet activation, and platelet degranulation. It indicated that the drug may inhibit platelet aggregation by binding to membrane proteins, affecting its energy utilization and activation. The pathway enrichment results also indicated that *R. procumbens* may play an antiplatelet aggregation role by inhibiting the key targets of the platelet activation signaling pathway, such as integrin α_IIb_β_3_. It was the intersection of all platelet-related pathways. Proteoglycans in cancer, PI3K–Akt signaling pathway, and so on also suggest that justicidin B may affect the expression and activity of cancer-related proteins by binding integrin α_IIb_β_3_.

Then, we use optical turbidimetry to measure the activity of three isolated blood absorption compounds. We found that justicidin B is the most active compound. Then, WB experiment verified this fact. It is well known that membrane proteins are less stable, and this has shown to have a massive impact on the MST (NT.LabelFree) experiment (Chasis and Mohandas, 1986). To identify optimal test and storage conditions for the membrane protein integrin α_IIb_β_3_, the protein was subjected to a thermal unfolding formulation screen of the Prometheus NT.48 (supplementary material). NanoTemper’s on-the-fly technology allows to measure 48 samples in parallel, providing more than 10 data points per minute. Formulation developments benefit from ultra-high resolution that are not compromised by aggregation but, at the same time, offer ease of use. Prometheus NT.48 results suggest that Hepes is the best detergent. The onset for ratio of Hepes-DDM is 46.1°C, which is higher than other buffers. This attempt was not reported before.

MST is a powerful technique to measure biomolecular interaction that are based on thermophoresis—the movement of molecules in a temperature gradient. This technique was reported to be highly sensitive such that it allows precise quantification of molecular interaction (Jerabek-Willemsen et al., 2014). Because of the influence of ligand fluorescence, we obtained unsatisfactory results, accompanied by relatively large errors. The results of the Prometheus NT.48 experiment showed that the protein stability was relatively good, and the labeled protein SD-test was qualified; hence, we experimented further with NT.115 and obtained better results. MST results suggest that the selected compound has potential molecular interaction with integrin α_IIb_β_3_. The Kd value of justicin B was 35.017 ± 5.9014 μm. These data suggest that justicin B has a good interaction with integrin α_IIb_β_3_. According to Seidel et al. (2013), the fitting curve may be either S-shaped or mirror S-shaped. The standard symbol of MST amplitude (change in normalized fluorescence) depends on the chemistry of the compound that is titrated, its binding site, and the conformational change induced upon binding. Justicin B shows a positive slope suggesting a strong conformational change induced upon complex formation. Probably its interaction plays a major role in conformational change. We speculate that it might interfere with the platelet aggregation mechanism by negatively influencing the conformational changes required for the integrin α_IIb_β_3_ activation.

Taken together, our results demonstrate that the ethyl acetate extract plays an anti-platelet aggregation role through integrin α_IIb_β_3_, and justicin B, which is the most active blood absorption compound, targets it. We believe that our findings would provide a better foundation for further understanding of the mechanism of *R. procumbens* intervention in platelet aggregation.

## Ethics Statement

This study was carried out in accordance with the recommendations of Animal Care and Use Committee of Institute of Materia Medica, China.

## Author Contributions

H-ZW conceived the study. H-ZW and Y-FY designed the study. S-TW performed the experiments and the data analysis, and wrote the manuscript. Y-FY, W-CX, BL, P-FH, W-PX, YS, Z-ZA, P-TY, and Z-TX revised the manuscript. All the authors read and approved the final version of the manuscript.

## Funding

This research was funded by the National Natural Science Foundation of China, grant number 31570343.

## Conflict of Interest Statement

The authors declare that the research was conducted in the absence of any commercial or financial relationships that could be construed as a potential conflict of interest.

## Supplementary Material

Figure S1 Thermal unfolding curves. **(A)** Thermal unfolding curves in presence of Hepes and pbs. **(B)** Thermal unfolding curves in presence of Ttis. Insets show the detergent dependence of the first unfolding transition midpoint (Tm1).
