# Supplementary figures and images for: A Novel Antiplatelet Aggregation Target of Justicidin B Obtained From Rostellularia Procumbens (L.) Nees
Source: Front Pharmacol. 2019 Jun 14;10:688. doi: 10.3389/fphar.2019.00688 (PMC6590258; doi:10.3389/fphar.2019.00688)

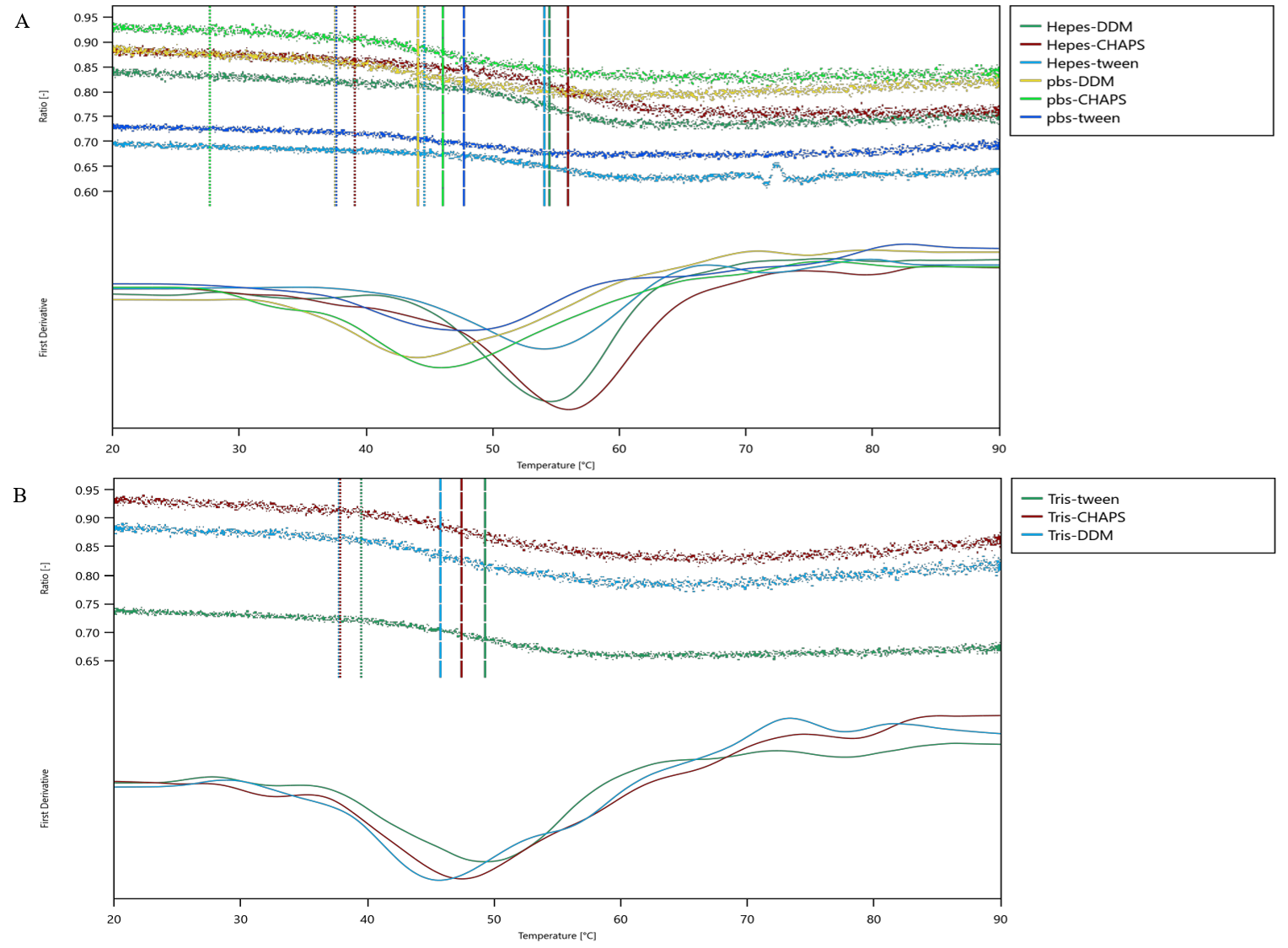

Supplement: Figure S1 — Thermal unfolding curves. (A) Thermal unfolding curves in presence of Hepes and pbs. (B) Thermal unfolding curves in presence of Ttis. Insets show the detergent dependence of the first unfolding transition midpoint (Tm1). [file Image_1.tif]
